# Spatiotemporal patterns and structural inequalities associated with the incidence of common workplace accidents in Brazil

**DOI:** 10.47626/1679-4435-2024-1334

**Published:** 2025-08-25

**Authors:** Claudio José dos Santos Júnior, Frida Marina Fischer

**Affiliations:** 1 School of Public Health, Universidade de São Paulo, São Paulo, SP, Brazil

**Keywords:** occupational accidents, socioeconomic factors, social determinants of health, ecological studies, spatial analysis., acidente de trabalho, fatores socioeconômicos, determinantes sociais da saúde, estudos ecológicos, análise espacial.

## Abstract

**Introduction:**

Despite extensive research on social determinants of health, little attention has been given to their association with occupational health indicators in Brazil, especially concerning workplace accident metrics.

**Objectives:**

To analyze the spatiotemporal pattern and the association between the occurrence of common workplace accidents and structural inequality indicators in Brazil.

**Methods:**

This ecological study investigated the incidence of common workplace accidents among Social Security beneficiaries from 2010 to 2019 and its association with structural inequality indicators. A temporal analysis using joinpoint regression was conducted, and spatial associations were investigated through Global and Local Moran’s I indices and corresponding maps.

**Results:**

The trend in the incidence of common workplace accidents showed a decline over the study period, with an annual percentage change of -3.3% (95% CI -4.3 to -2.2; p = 0.001). A moderate negative correlation was found between accident incidence and the Theil-L Index (Moran’s I = -0.541; p = 0.001), Gini Index (Moran’s I = -0.544; p = 0.001), and Social Vulnerability Index (Moran’s I = -0.558; p = 0.001). Conversely, a positive correlation was observed with the Municipal Human Development Index (Moran’s I = +0.542; p = 0.002).

**Conclusions:**

In summary, there was a decrease in the incidence of common workplace accidents in Brazil, and a spatial association was found between this indicator and inequalities at the territorial level.

## Abstract (pt)

**Introdução:**

Apesar da vasta pesquisa sobre os determinantes sociais em saúde, pouco se explorou a associação destes com indicadores de saúde do trabalhador no Brasil, especialmente em relação às métricas de acidentes do trabalho.

**Objetivos:**

Analisar o padrão espaçotemporal e a associação da ocorrência de acidentes típicos do trabalho com indicadores de iniquidades estruturais no Brasil.

**Métodos:**

Trata-se de um estudo ecológico que analisou a incidência de acidentes típicos do trabalho em segurados da Previdência Social, de 2010 a 2019, e a associação desse indicador com indicadores de iniquidades estruturais. Foram realizadas análise temporal por *joinpoint* e investigação de associações espaciais através do Índice de Moran Global e Local e de seus mapas.

**Resultados:**

A tendência da incidência de acidentes típicos do trabalho ao longo do período foi decrescente, com variação percentual anual de -3,3% (intervalo de confiança de 95%: -4,3 a -2,2; p = 0,001). Houve correlação negativa moderada desse indicador com os índices de Thei-L (Moran’s I = -0,541; p = 0,001), Gini (Moran’s I = -0,544; p = 0,001) e Índice de Vulnerabilidade Social (Moran’s I = -0,558; p = 0,001), e positiva com o Índice de Desenvolvimento Humano Municipal (Moran’s I = +0,542; p = 0,002).

**Conclusões:**

Evidenciou-se, em síntese, diminuição na incidência de acidentes típicos do trabalho no Brasil e associação espacial desse indicador com iniquidades em nível territorial.

## INTRODUCTION

An workplace accident (WPA) is defined as an incident occurring during work that results in harm to the worker - whether in the form of bodily injury or functional impairment leading to death or a permanent or temporary loss or reduction of work capacity.^1^ Its classification requires establishing a causal relationship between work, the accident, and the resulting injury. Among the different types of WPAs, the most notable are common workplace accidents (CWPAs), occupational diseases and work-related illnesses, including atypical accidents, commuting accidents, and other legally equivalent conditions.^1^

Work-related accidents account for approximately 2.93 million deaths worldwide, of which 330,000 correspond to CWPAs and 2.6 million to occupational diseases, along with 395 million nonfatal work injuries.^2^ In Brazil, in 2022, the Ministry of Health reported 277,322 cases of WPAs, including 3,815 fatal work injuries.^3^ Among insured workers under the General Social Security System (*Regime Geral de Previdência Social*, RGPS), 648,366 WPA cases were recorded in the same period, of which 418,684 were CWPAs, 125,505 were commuting accidents, 27,659 were occupational diseases, and 76,518 were due to a technical causal relationship, with 2,842 being fatal.^4^

Global estimates suggest that the economic burden of these incidents ranges from 1.8% to 6% of a country’s gross domestic product (GDP).^5^ The main associated costs include sick leave benefits, disability pensions, survivor benefits, rehabilitation, hospitalizations, medications, compensations, and indirect costs such as lost income, premature deaths, and reduced labor productivity.^6,7^

In lowand middle-income countries, the impact of WPAs is significantly greater.^5^ These regions often face challenges such as inadequate health care infrastructure, ineffective enforcement of occupational health and safety regulations, and precarious work conditions.^7^

Structural determinants - such as the distribution of power and income, access to health care, housing, and education - play a crucial role in shaping population health and contributing to health inequities.^8,9^ Despite extensive research on these determinants, little attention has been given to their association with occupational health indicators in Brazil, particularly in relation to WPA metrics.

This study aimed to analyze the spatiotemporal pattern and the association between CWPA occurrence and indicators of structural inequities in Brazil.

## METHODS

This ecological study used secondary data from the Brazilian Ministry of Social Security and the United Nations Development Programme (UNDP) to analyze the incidence of CWPAs in Brazil over a 10-year period (2010-2019) and its association with structural determinants of health.

Data were collected from the Historical Occupational Accident Database of the Statistical Yearbook of Occupational Accidents (AEAT InfoLog) and the UNDP Human Development Atlas.

The dependent variable was the CWPA incidence rate among RGPS-insured workers per federative unit (FU). Annual incidence rates per FU were calculated using the following equation: incidence = (number of CWPA in the reference year / average number of employment contracts in the reference year) × 1,000.

This study focused on CWPAs due to the variable pathophysiology of work-related accidents. CWPAs are sudden and unforeseen events occurring during work activities. In contrast, occupational diseases develop gradually due to work processes, and commuting accidents are not directly related to the workplace but rather to the worker’s travel between home and work. Because of their sudden and visible nature, CWPAs are more likely to be easily identified and reported.

The independent variables included four socioeconomic inequality indicators: the Theil-L Index, Gini Index, Municipal Human Development Index (MHDI), and Social Vulnerability Index (SVI). More information about these indicators can be accessed on the UNDP website (http://www.atlasbrasil.org.br/).

To assess the temporal trend of CWPA incidence among RGPS-insured workers, we applied a segmented log-linear regression model using joinpoint regression. Temporal trends were described using the annual percent change (APC) and its 95% CI. Trends were considered statistically significant if the APC had a p-value < 0.05 and the 95% CI did not include 0. A positive and significant APC indicated an increasing trend, while a negative and significant APC indicated a declining trend.^10^ Nonsignificant trends were classified as stable, regardless of the APC values. Analyses were conducted on the Joinpoint Regression software, version 5.0.2.

A univariate Global Moran’s I Index was used to examine spatial autocorrelation and identify patterns in the incidence of CWPAs and inequality indicators. This analysis estimates spatial correlation, with values ranging from -1 to +1, where values close to 0 indicate no spatial autocorrelation. If spatial autocorrelation was detected, we performed a local autocorrelation analysis using the Local Moran’s I Index (Local Indicators of Spatial Association [LISA]), which classified areas into high-high (HH), low-low (LL), high-low (HL), and low-high (LH) quadrants, considering p < 0.05 as significant. Additionally, we examined the association between CWPA incidence and inequality indicators using bivariate Global and Local Moran’s I indices.^11^

Maps and spatial analysis were performed using GeoDa software, version 1.22. Since we used publicly available, aggregated data, approval by an ethics committee was not required.

## RESULTS

**Figure 1:**
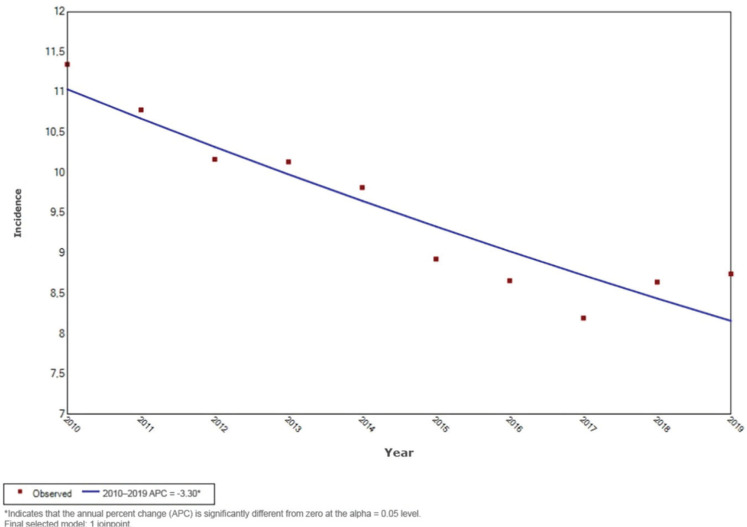
Historical series and temporal trend of the incidence of common workplace accidents per 1,000 employment contracts in Brazil, 2010-2019.

From 2010 to 2019, a total of 3,956,045 CWPAs were reported among RGPS-insured workers in Brazil, resulting in an average incidence of 9.54 cases per 1,000 employment contracts. During this period, a declining trend was observed, with an APC of -3.3% (95% CI: -4.3 to -2.2; p = 0.001) (Figure 1).

At the FU level, most states and the Federal District also showed a declining trend, except for Mato Grosso, Santa Catarina, and Tocantins, where no significant change was observed over time (p ≥ 0.05). No FU exhibited an increasing trend in CWPA incidence (Table 1).

**Table 1:** Temporal trend of the incidence of common workplace accidents per 1,000 employment contracts by FU, Brazil, 2010-2019

FU	APC (%)	95% Cl	p-value	Trend
Acre	-4.7	-6.5 to -2.9	< 0.001	Declining
Alagoas	-13.3	-15.7 to -10.9	0.004	Declining
Amapá	-6.8	-10.8 to -2.6	0.006	Declining
Amazonas	-4.1	-6.3 to-1.9	0.007	Declining
Bahia	-3.8	-5.4 to -2.2	0.001	Declining
Ceará	-2.0	-3.8 to -0.2	0.017	Declining
Distrito Federal	-2.8	-4.3 to -1.2	0.011	Declining
Espirito Santo	-1.4	-2.4 to -0.3	0.016	Declining
Goiás	-1.7	-3.2 to -0.2	0.010	Declining
Maranhão	-4.9	-8.2 to -1.5	0.013	Declining
Mato Grosso	-1.5	-2.9 to 0.0	0.061	Stable
Mato Grosso do Sul	-1.4	-2.4 to -0.3	0.017	Declining
Minas Gerais	-3.1	-4.7 to -1.5	< 0.001	Declining
Pará	-5.3	-6.6 to -3.9	< 0.001	Declining
Paraiba	-6.0	-7.5 to -4.5	< 0.001	Declining
Paraná	-2.1	-3.7 to -0.4	0.009	Declining
Pernambuco	-6.9	-9.9 to -3.8	0.035	Declining
Piaui	-3.7	-6.4 to -0.9	0.017	Declining
Rio de Janeiro	-3.1	-4.2 to -2.0	< 0.001	Declining
Rio Grande do Norte	-5.9	-7.6 to -4.1	< 0.001	Declining
Rio Grande do Sul	-1.3	-2.4 to -0.2	0.029	Declining
Rondônia	-5.0	-8.0 to -1.9	0.006	Declining
Roraima	4.2	0.1 to 8.5	0.048	Declining
Santa Catarina	-0.4	-3.4 to 2.7	1.000	Stable
São Paulo	-2.9	-4.7 to-1.1	0.019	Declining
Sergipe	-4.9	-7.4 to -2.5	0.043	Declining
Tocantins	-0.5	-2.1 to 1.1	0.486	Stable

APC = annual percent change; FU = federative unit.

**Figure 2:**
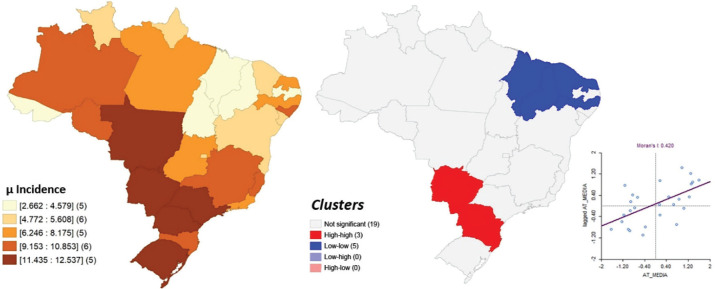
Spatial distribution of the average incidence of typical workplace accidents per 1,000 employment contracts and spatial autocorrelation of this variable, according to the Local Moran’s I Index (LISA) univariate, Brazil, 2010-2019.

**Figure 3:**
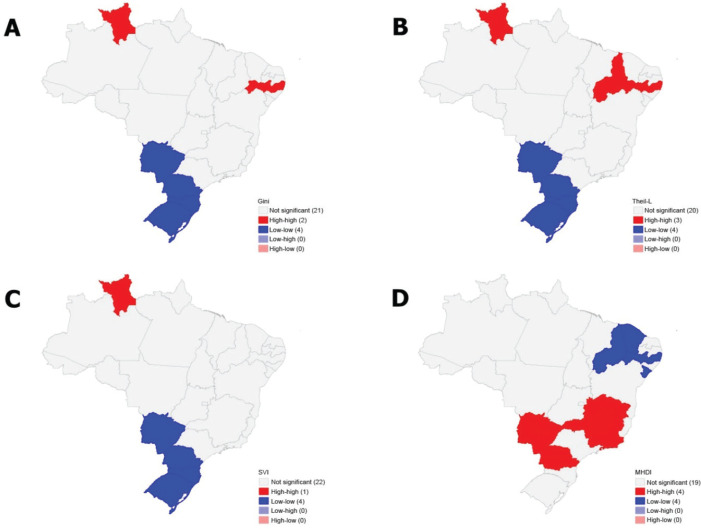
Spatial autocorrelation of socioeconomic inequality indicators according to the Local Moran’s I Index (LISA) univariate, Brazil, 2010. A) Theil-L; B) Gini; C) Social Vulnerability Index (SVI); D) Municipal Human Development Index (MHDI).

Disparities were identified in the distribution of CWPA incidence per 1,000 employment contracts across Brazil (Figure 2). FUs in the South, Southeast, and Center-West regions recorded the highest accident rates, which coincided with lower inequality levels according to the Theil-L, Gini, SVI, and MHDI indices. In contrast, states in the North and Northeast regions exhibited lower CWPA incidence rates but higher socioeconomic disparities (Figure 3).

The Global Moran’s I Index revealed a moderate positive spatial autocorrelation for CWPA incidence in Brazil, with a value of 0.420 (p ≤ 0.05). Two clusters were identified among the 27 FUs analyzed: one HH, comprising three FUs, and one LL, comprising five FUs (Figure 2). In the univariate analysis, a moderate spatial autocorrelation was observed for all four socioeconomic indicators (Figure 3).

**Figure 4:**
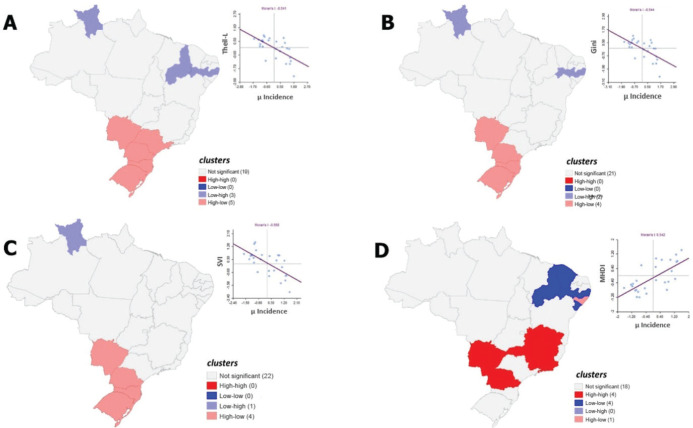
Spatial correlation of the average incidence of common workplace accidents per 1,000 employment contracts and socioeconomic inequality indicators according to the Local Moran’s I Index (LISA) bivariate, Brazil, 2010-2019. A) Theil-L; B) Gini; C) Social Vulnerability Index (SVI); D) Municipal Human Development Index (MHDI).

In the bivariate analysis, the bivariate Local Moran’s I maps identified statistically significant clusters between CWPA incidence and socioeconomic inequality indicators across different FUs in Brazil (Figure 4).

The average incidence of CWPAs among RGPSinsured workers showed a moderate negative correlation with the Theil-L Index (Moran’s I = -0.541; p = 0.001), Gini Index (Moran’s I = -0.544; p = 0.001), and SVI (Moran’s I = -0.558; p = 0.001), while a moderate positive correlation was observed with the MHDI (Moran’s I = 0.542; p = 0.002) (Table 2).

**Table 2:** Bivariate spatial autocorrelation of the average incidence of common workplace accidents (CWPA) per 1,000 employment contracts and socioeconomic inequality indicators, Brazil, 2010-2019

Bivariate Local Moran’s I	Theil-L	Gini	SVI	MHDI
CWPA Incidence	I = -0.541	I = -0.544	I = -0.558	I = 0.542
	p-value = 0.001	p-value = 0.001	p-value = 0.001	p-value = 0.002
	z-score = -4.381	z-score = -4.434	z-score = -4.790	z-score = 4.827

I = Local Moran’s I Index (Local Indicators of Spatial Association [LISA]); MHDI = Municipal Human Development Index; SVI = Social Vulnerability Index.

## DISCUSSION

This study explored the spatiotemporal patterns of CWPA occurrence in Brazil, focusing on RGPS-insured workers and the association of these incidents with structural inequality indicators.

The findings revealed a declining trend in CWPA incidence nationwide and across 24 FUs between 2010 and 2019, aligning with existing literature.^12-15^

Significant regional disparities were observed in the concentration of CWPA cases, with higher notification rates in Southern, Southeastern, and Center-West FUs.

A positive spatial autocorrelation was detected for CWPA incidence, indicating the formation of spatial clusters with neighboring regions presenting similar incidence patterns.^11^ This evidence suggests that CWPAs are spatially concentrated, highlighting the need for prevention policies that account for this geographic dimension in their design.^16^

The moderate negative correlation between CWPA incidence and inequality indices (Theil-L and Gini) as well as the SVI suggests that territories with higher inequality and social vulnerability reported lower CWPA incidence rates. Similarly, the positive correlation between CWPA incidence and the MHDI indicates that regions with higher human development had higher CWPA incidence rates.

In general, previous studies have typically reported a negative correlation between development indicators and WPA rates.^17-19^ However, the results of this study suggest a different dynamic, where higher inequality and social vulnerability were associated with lower CWPA incidence rates, while more developed and less vulnerable areas exhibited higher CWPA incidence. A possible explanation for this pattern is that in highinequality areas, limited resources and restricted access to health care services may play a crucial role in underreporting these incidents to Social Security.

Higher economic development is also associated with better working conditions, stricter occupational safety enforcement, and a workforce that is more aware of and educated on occupational risks.^5,7,17,19-22^ Kahraman et al.^22^ reported a 1.1% reduction in fatal WPAs for every 1% increase in national income. Similarly, Moniruzzaman & Andersson^23^ found that mortality rates in high-income countries decreased as economic development improved. Li et al.^19^ highlighted that increased investment in scientific research and education leads to higher-quality workers and the training of occupational safety professionals, a conclusion supported by negative indirect correlations between socioeconomic indicators (research investment, education spending, and wages) and WPA incidence.^19^

A possible explanation for the identified clusters is the disparities resulting from unequal economic growth across Brazil. The identified clusters, in both univariate and bivariate analyses, underscore Brazil’s regional disparities, which are reflected in the distribution of national GDP: 52.3% in the Southeast, 17.3% in the South, and 10.3% in the Center-West. Conversely, the North and Northeast account for only 20.1% of the GDP despite high population density in the Northeast.^24^ Additionally, 62.4% of municipalities in the Northeast and 42.4% in the North lack sufficient resources for self-sustainability, compared to only 5.9% in the South, 11.4% in the Center-West, and 15% in the Southeast.^25^ The concentration of economic assets, institutional capacity, and well-being opportunities is significantly higher in the Southeast and South, where the country’s main cities and financial hubs are located. This imbalance influences regional human, institutional, and environmental development levels.

Consistent with our study, Mascarenhas et al.^26^ also reported that WPA rates in Brazil are highest in regions with greater economic development. The authors emphasized that these areas show a higher proportion of emergency care visits for work-related injuries, likely due to the higher concentration of industries and maintenance services.^26^ The high number of rural workers, driven by agribusiness, is another factor contributing to the elevated accident rates in FUs where agribusiness is more prominent.^15^

Furthermore, the formalization of the labor market may be increasing the statistical visibility of CWPAs. In contrast, in regions where informal work predominates, underreporting is likely, leading to an artificially lower number of recorded cases. The rise in informal employment in Brazil, which increased from 39.8% in 2012 to 43.4% in 2019, may have influenced the results of this study.^27^ The informal work environment poses an additional risk, as many workers lack access to safety standards and protections enjoyed by formal employees. This includes lack of proper training, absence of protective equipment, and a workplace culture that tolerates hazardous practices.^27,28^ In addition, when informal workers suffer accidents, even if they contribute to Social Security as self-employed individuals, these do not fall under the legal definition of WPAs, as outlined in Article 19 of Law 8.213/1991^1^. As a result, the true incidence of CWPAs in regions with high informal labor prevalence, such as the North and Northeast, is likely to be underestimated.

In addition to the factors already discussed, a key issue in Brazil’s occupational health system is underreporting. This leads to an underestimation of the true CWPA incidence in the country.^29^ Consequently, many CWPAs, especially less severe cases, may go unreported. Several factors contribute to this reality, including insufficient workplace inspections, lack of infrastructure for adequate workplace monitoring, limited worker awareness and education, failure to recognize the severity of WPAs, a workplace that tolerates injuries, and fear of retaliation or job loss.

Furthermore, in some regions, work organization, socioeconomic conditions, and political factors may also contribute to concealing or neglecting these incidents. Additional challenges include limited access to health care services and worker protection agencies, communication barriers and data collection difficulties in remote or underdeveloped areas, and limited infrastructure and resources. As a result, the true CWPA incidence is likely significantly higher than reported. Moreover, the quality of accident reports varies across the country, potentially affecting data accuracy. This study focused exclusively on CWPAs reported to Social Security, excluding statutory workers, military personnel, and those outside the RGPS. Other types of WPAs were also not considered. However, despite these limitations, this study was based on an official database, widely used in occupational and Social Security research, ensuring reliable and comparable results.

## CONCLUSIONS

The results demonstrated a declining trend in the incidence of CWPAs among RGPS-insured workers over the study period. Additionally, a significant association was observed between CWPA incidence and the four structural inequality indicators examined (Theil-L Index, Gini Index, MHDI, and SVI), along with the formation of spatial clusters and neighboring regions with similar incidence patterns.

These findings underscore the need for WPA prevention strategies to be integrated into broader regional disparity reduction efforts in Brazil. Therefore, factors such as regional inequalities, human development, and social vulnerability must be considered by the Brazilian government in the design of public policies related to occupational safety and health.
